# Supplementary material for: SARS-CoV-2 detection in multi-sample pools in a real pandemic scenario: A screening strategy of choice for active surveillance
Source: PLoS One. 2022 Apr 1;17(4):e0266408. doi: 10.1371/journal.pone.0266408 (PMC8975135; doi:10.1371/journal.pone.0266408)
Supplement: Attachment — Submitted filename: Response to Reviewers.docx [file pone.0266408.s002.docx]

All the authors and I wish to take this opportunity to acknowledge the editor and the reviewers for their constructive comments and valuable recommendations. We have revised the manuscript on our R1 version according to the concerns, and have resubmitted it online. Our responses are listed below in blue font.

**ACADEMIC EDITOR**

1. Check the first paragraph of the introduction as there is no references. Is it your own sentences?

This paragraph was written based on our own experience and the situation that many countries in the world went through. The corresponding citations supporting these sayings have been added.

2. Replace “in order to” with “to” throughout the text

In line 90 of manuscript, “in order to” was replaced by “to”.

3. Check spaces and punctuation throughout the text

Following the editor's comment, spaces and punctuation were checked through the manuscript.

4. P letter for statistical value should be uppercase-italic face

Now, the *P* letter for statistical value now have the indicated format.

5. Figs captions should not be incorporated in the text. They should be at the bottom of the corresponding one.

At the editor’s request, figure captions were moved from the body text to the bottom of the manuscript.

6. Avoid using “we, our”. Use impersonal phrasing throughout the text

As suggested, most of the personal phrases in the manuscript were changed to impersonal phrases.

7. Study strengths and limitations should be in a separate section, headed as addressed. It should be ahead of the conclusion. Start with the strength of the study, followed by limitations.

The discussion section has been reordered according to the editor’s suggestion. Now, the first part exposes the strengths of the pooling strategy followed by the limitations, both under the corresponding subtitle.

8. The conclusion should be in a separate section. What are the clinical relevance and future perspective? Add this to the conclusion section.

Now the conclusion is in a separate section after the discussion.

9. Proofread the text for grammar and syntax errors

Based on the editor’s comment, English, grammar, and syntax were checked throughout the manuscript.

**Reviewer #1:**

1. According to the reviewer’s comment: *Line 108 currently states: "These oropharyngeal swab samples were pooled together into 5 samples based on the order number, without mixing samples from different institutions."*
   *This would be more clearly written as "Each group of 5 sequentially obtained samples was pooled, without mixing samples from different institutions."*

We thank the reviewer’s advice and following it, the paragraph was changed according to the suggestion (line 102).
